# A Machine Learning Decision Support System (DSS) for Neuroendocrine Tumor Patients Treated with Somatostatin Analog (SSA) Therapy

**DOI:** 10.3390/diagnostics11050804

**Published:** 2021-04-28

**Authors:** Jasminka Hasic Telalovic, Serena Pillozzi, Rachele Fabbri, Alice Laffi, Daniele Lavacchi, Virginia Rossi, Lorenzo Dreoni, Francesca Spada, Nicola Fazio, Amedeo Amedei, Ernesto Iadanza, Lorenzo Antonuzzo

**Affiliations:** 1 Computer Science Department, University Sarajevo School of Science and Technology, 71210 Sarajevo, Bosnia and Herzegovina; jasminka.hasic@ssst.edu.ba; 2 Medical Oncology Unit, Careggi University Hospital, Largo Brambilla 4, 50134 Florence, Italy; serena.pillozzi@unifi.it (S.P.); daniele.lavacchi@yahoo.it (D.L.); virginiarossi89@gmail.com (V.R.); lorenzodreoni@gmail.com (L.D.); lorenzo.antonuzzo@unifi.it (L.A.); 3 Department of Information Engineering, University of Florence, Via S. Marta 3, 50139 Florence, Italy; rachele.fabbri@stud.unifi.it (R.F.); ernesto.iadanza@unifi.it (E.I.); 4 Division of Gastrointestinal Medical Oncology and Neuroendocrine Tumors, European Institute of Oncology, IEO, IRCCS, Via Ripamonti 435, 20141 Milan, Italy; alice.laffi@ieo.it (A.L.); francesca.spada@ieo.it (F.S.); nicola.fazio@ieo.it (N.F.); 5 Department of Experimental and Clinical Medicine, University of Florence, Largo Brambilla 3, 50134 Florence, Italy

**Keywords:** neuroendocrine tumors, machine learning, prognostic factors, predictive biomarkers, somatostatin analogs, random forest classifier

## Abstract

The application of machine learning (ML) techniques could facilitate the identification of predictive biomarkers of somatostatin analog (SSA) efficacy in patients with neuroendocrine tumors (NETs). We collected data from 74 patients with a pancreatic or gastrointestinal NET who received SSA as first-line therapy. We developed three classification models to predict whether the patient would experience a progressive disease (PD) after 12 or 18 months based on clinic-pathological factors at the baseline. The dataset included 70 samples and 15 features. We initially developed three classification models with accuracy ranging from 55% to 70%. We then compared ten different ML algorithms. In all but one case, the performance of the Multinomial Naïve Bayes algorithm (80%) was the highest. The support vector machine classifier (SVC) had a higher performance for the recall metric of the progression-free outcome (97% vs. 94%). Overall, for the first time, we documented that the factors that mainly influenced progression-free survival (PFS) included age, the number of metastatic sites and the primary site. In addition, the following factors were also isolated as important: adverse events G3–G4, sex, Ki67, metastatic site (liver), functioning NET, the primary site and the stage. In patients with advanced NETs, ML provides a predictive model that could potentially be used to differentiate prognostic groups and to identify patients for whom SSA therapy as a single agent may not be sufficient to achieve a long-lasting PFS.

## 1. Introduction

Neuroendocrine tumors (NETs) arise from neuroendocrine cells distributed throughout the body. They consist of a wide family of tumors that includes the pancreatic NET (panNET) and gastrointestinal (GI) NET and also neoplasms from other origin sites [1]. The incidence of NETs in the United States was 6.98 cases per 100,000 people in the year 2004 and it increased from 1975 to 2008. The reasons for this rise are unclear although the improvement of diagnosis and classification seem to be two of the major factors [2,3]. Patients with NETs may or may not have symptoms attributable to hormonal hypersecretion (“functional” or “non-functional” tumors) [4]. Most NETs are sporadic with unknown risk factors whereas rare cases are related to inherited syndromes including multiple endocrine neoplasia (MEN) 1 and 2, von Hippel–Lindau (VHL) disease, tuberous sclerosis (TSC) complex and neurofibromatosis (NF) 1 [5,6,7]. In addition to the site of origin, NETs are generally subclassified by histologic characteristics based on a tumor differentiation and proliferation index [8]. Several studies have confirmed that an increased mitotic rate and a high Ki67 index are associated with a more aggressive clinical behavior with a consequently worse prognosis [9,10,11,12,13,14]. NETs are staged according to the eighth edition of the AJCC tumor (T), node (N) and metastasis (M) staging system [15]. The association of the tumor stage with the prognosis has been confirmed in analyses of the SEER database and the National Cancer Database [16,17,18,19,20]. Nevertheless, in addition to information on the histologic classification and stage, other factors are under study to verify a potential prognostic role such as the margin status (positive or negative) and the presence of a vascular or perineural invasion [21,22], the serum level of Chromogranin A [23], the overexpression of the mammalian target of rapamycin (mTOR) [24], mutations or the loss of expression in the cyclin-dependent kinase inhibitor CDKN1B (p27) [25,26] and circulating tumor cells (CTCs) [27]. As most NETs overexpress high-affinity receptors for somatostatin, mainly subtype 2 [28], the use of somatostatin analogs (SSAs, octreotide LAR and lanreotide depot) can be effective for both syndrome control and tumor growth control [29]. The evidence for the use of octreotide LAR (30 mg/4 w) is based on the results of the PROMID study in metastatic midgut NETs, which showed a median time to tumor progression (TTP) of 14.3 and 6 months in the octreotide LAR and placebo groups, respectively [30,31]. Subsequently, the CLARINET study randomized 204 patients with locally advanced or metastatic non-functioning pancreatic or intestinal NETs [32] and showed that treatment with lanreotide for two years resulted in an improvement in the PFS over a placebo (32.8 vs. 18 months) [33]. Although these two trials confirmed with a high level of evidence the antiproliferative effect of SSAs, after decades of a very poor level of evidence for this, no clear predictive factors came up from the studies to realize which NETs would benefit most from an SSA [34].

Recent scientific breakthroughs and technical developments have expanded our cancer understanding and changed approaches to diagnosis and treatment resulting in more accurate, predictive, preventive and personalized health care tailored to the individual patient. Consequently, the big data revolution has provided an opportunity to mine such a large dataset by implementing artificial intelligence (AI) and machine learning (ML) algorithms. In addition, personalized medicine aims to revolutionize healthcare with its main purpose of providing the proper patient with the proper medication at the proper time and dosage and thereby improving the quality of life and, finally but importantly, helping to reduce the healthcare cost.

AI and ML, which can be briefly defined as technologies enabling computers to make successful predictions using past experiences, have shown an impressive development recently with the help of the great increase in the processing power and storage capacity of computers. ML methods have been widely employed in bioinformatics [35,36] but recently also in the health area and especially in support of cancer management including diagnosis, prognosis and treatment.

Several studies, for example, have attempted to use deep learning (DL) to help identify dysplasia and early esophageal cancer [37] while different AI models have been developed to evaluate different aspects of gastric cancer such as the diagnosis or prognosis [38]. In addition, DL models have been used in breast cancer to identify potential diagnostic biomarkers [39] and to improve the accuracy in the histologic classification [40] or diagnosis [41].

Finally, and notably, in a recent study Goehler et al., using data of 64 NET patients, constructed a DL algorithm that discovered liver metastases, co-registered the detected lesions and then assessed the interval change in the cancer burden between two multiparametric liver MRI examinations [42].

Starting from these premises, we selected a homogeneous population of gastroenteropancreatic (GEP) NETs treated with a first-line SSA with an antiproliferative goal and focused our study to identify through ML the clinical and/or biological factors predicting the clinical outcomes.

## 2. Results

### 2.1. Cohort of Patients

A total of 74 patients were enrolled; in detail, 30 patients with a pancreatic NET and 44 patients with a gastrointestinal NET. Of these, 57 patients were less than 70 years of age, 17 were 70 or more years of age and the median age was 68 years. The total number of men was 45 and the total number of women was 29. A total of 97% of the cohort population had a metastatic disease, only 3% had a locally advanced disease and the tumor had a hormonal hypersecretion in 30% of the cases. G1 tumors were present in 35% of the patients whereas 62% of the patients had G2 tumors. The grade had not been assessed in two patients (3%). Considering the mitotic rate, the Ki67 was <2% in 26% of the cases, 2–20% in 69% of the cases, ≥20% in 2.6% of the cases and not assessed in two cases (2.5%). The primary tumor was in site in 42% of the patients while 58% underwent surgery and had a local or distant recurrence of the disease. A total of 62% of the population had a single metastatic site, 35% had more than one metastatic site while 3% had no distant metastases. Metastases were localized in the liver in the majority of the cases (85%) followed by the lung in 7% of the cases and bones in 4% of the cases. Of the cohort, 46% of the patients received a treatment with a lanreotide injection every 28 days and 54% had an injection of octreotide LAR every 28 days. A severe adverse event (G3–G4) related to the treatment occurred in only one patient. The PFS was more than 12 months from the beginning of the treatment in 72% of the patients and more than 18 months in 58% of the cases while it was not evaluated in four patients (5%) because they had started therapy for less than 12 months. The dataset characteristics are depicted in Table A1.

### 2.2. Data Cleaning

Before proceeding with the data analysis, the dataset was examined. As four samples did not have the information of whether that person progressed either after 12 or 18 months (or not at all), those samples were removed from the dataset. An additional two samples had data missing for the grade and Ki67 features but as all of the other features were present as well as the progression outcomes, we decided to retain those samples and replace the missing values with the average for those features amongst all of the other samples.

Additionally, we engineered one feature and that was the current age in years. The date of birth was then not considered as a feature. The “Performance status (ECOG)” feature had a value of zero for all but one sample and it was removed from the dataset as it could not contribute to the analysis.

In the end, the analyzed dataset had 70 samples and 15 features. The outcomes of those 70 patients were: 17 patients progressed after 12 months, an additional 10 progressed after 18 months and the remaining 43 patients had no disease progression.

### 2.3. Classification Models

Based on the available outcomes for the patient data, we initially decided to develop the following classification models:

- Model 1: predicts whether the patient will progress after 12 months;
- Model 2: predicts whether the patient will progress after 18 months;
- Model 3: predicts whether the patient will progress either after 12 or 18 months or not at all.

The first two models had two possible outcomes while the third one had three outcomes. For the first two models a random guess would be correct half of the time (50%) on average while for the third model a random guess would be correct a third of the time (around 33%).

### 2.4. Imbalance Analysis of the Dataset

**Figure 1:**
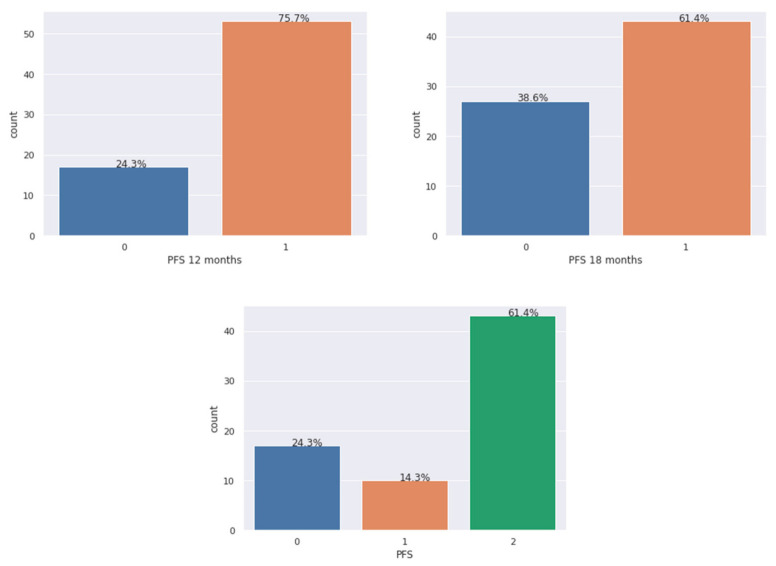
Plots of the count of samples per class. From the left: model 1–PFS 12 months, model 2—PFS 18 months, model 3—PFS.

Table A2 summarizes the sample counts for each of the studied models. As we can see, the outcomes in our datasets were not very well balanced as there was a large difference in their counts. Furthermore, Figure 1 presents the counts in the form of a graph: each bar represents the number of samples available for that class for the three above-mentioned models. On top of each bar, the percentage of samples per class is given. In ML, these types of situations can lead to poor performance of the minority outcome prediction. The description of all of the outcomes for the three studied models can be found in Table 1.

### 2.5. Fixing the Data Imbalance

As analyzed in the previous section, the dataset was not balanced for all of the three models considered: progression at 12 months, progression at 18 months and progression either at 12 or 18 months.

**Figure 2:**
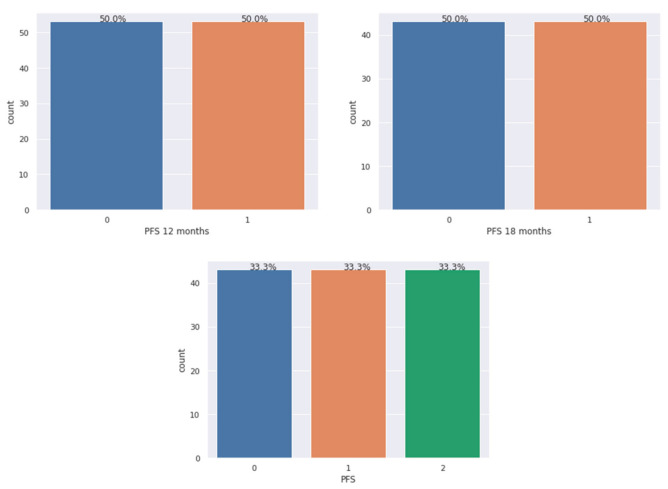
Balanced dataset after applying SMOTE. From the left: model 1—PFS 12 months, model 2—PFS 18 months, model 3—PFS.

The imbalance of the dataset was fixed with SMOTE, an oversampling method for creating synthetic examples of the minority classes. The results of the oversampling process on our dataset are shown in Figure 2.

### 2.6. Feature Selection

The results of the FS process are shown in Table 2. A cutoff threshold of three was adopted: only the features with a score greater or equal to 3 were included in the final set. The features and their respective score are reported.

### 2.7. ML Algorithms

The features identified in Table 2 were used to train 10 different ML algorithms for each model. Table 3 summarizes the performance of ten different ML algorithms. In addition to the accuracy score, further metrics (precision, recall and F1-score) were reported for each studied class (progressed and progression-free patients). All algorithms were run using the 10-fold cross-validation.

### 2.8. Hyperparameter Tuning

The results in Table 3 corresponded with running algorithms with the default parameter settings. For all of the algorithms that exhibited an accuracy over 70% we performed hyperparameter tuning. Those included logistic regression, Multinomial NB, MLP, SVC and K-Nearest Neighbors classifiers. The range and domain of the tested parameters can be found in the Appendix A. In addition, ten random seeds were generated and average accuracies were calculated for those five algorithms. A summary of the calculated accuracies can be found in Table 4. After the hyperparameter tuning, the Multinomial NB algorithm still exhibited the highest accuracy. It also showed the smallest improvement as the best parameters were the closest to the default values.

To examine the statistical significance in the performance differences between the three models, an ANOVA statistical test was used; a statistical *t*-test was also used to establish significance in the performance differences for pairs of models. Model 1 exhibited the greatest accuracy (*p* < 0.01) while Model 2 was the least accurate (*p* < 0.01). This performance was repeated for all five algorithms used in this section. The statistical tests were run both on accuracies of all single algorithms and on aggregated runs of all five algorithms, confirming the statistically significant superiority of Model 1 (*p* < 0.01).

## 3. Discussion

In order to identify clinical factors that may predict outcomes in patients who received SSAs as a first-line treatment for a GEP NET, we used ML algorithms and developed three models with two or three possible outcomes. The classification algorithm that performed the best was Multinomial Naïve Bayes, which in general performs well for classifications with discrete features. Overall, the factors that mainly influenced PFS included age, the presence of liver or nodal metastases, the primary site, the tumor grade and Ki67. These results were consistent with previous studies although this field is still little explored. Collecting data from 535 patients, of whom 438 were from the R-GETNE training cohort and 97 from The Christie NHS Foundation Trust of Manchester (external validation subset), Carmona-Bayonas et al. developed an accelerated failure time model to predict PFS in patients who received a first-line SSA for an advanced, well-differentiated NET. Overall, PFS was 28.7 months and overall survival (OS) was 85.9 months. The study identified nine factors associated with PFS: primary tumor location, Ki67, neutrophil-to-lymphocyte ratio, alkaline phosphatase, the extent of liver involvement, bone and peritoneal metastases, the deterioration of the performance status during treatment and symptoms at the baseline15. In another retrospective cohort of patients treated with octreotide LAR for advanced NETs, Laskaratos et al. identified the pancreatic primary tumor location, liver metastases and intermediate grade tumors as predictors of a poor TTP. In contrast, age, extra-hepatic metastases, mesenteric desmoplasia, previous resection and functionally active disease were not associated with the treatment response. Additionally, the subgroup analysis from the CLARINET trial showed no difference in the therapeutic effects of lanreotide on PFS according to age (≤65 years vs. >65 years) while sex, age, ethnicity, geographical region, time since diagnosis, Ki67 percentage, tumor grade, chromogranin A level, prior chemotherapy and prior surgery were not associated with PFS.

If confirmed in future studies, the current findings provide a rationale for differentiating patients for whom an SSA single agent may not be sufficient to achieve a long-lasting PFS. Nuclear medicine and molecular imaging features may be incorporated into these algorithms as separate prognostic markers to help distinguish the prognosis.

Starting with the initial dataset, we firstly needed to formulate a classification that could be achieved with all of the constraints that were embedded in it. Given its size and the number of samples containing all of the different outcomes, an ML classifier could be built to answer the following question: “Is the patient going to progress within 18 months?”

The classification of an unknown sample with such a classifier was either “progressed” or “progression-free”. We engineered one feature from our dataset (age in years) that turned out to be the feature of highest importance.

We applied ten different ML algorithms (described in Section 4.4). The performance of these algorithms with their default parameters is summarized in Table 3. We reported four different metrics for each algorithm (accuracy, precision, recall and F1-score). Furthermore, we applied hyperparameter tuning on the five best performing algorithms from Table 3 and those results are summarized in Table 4. The multi-layer perceptron (MLP) and support vector machine classifiers (SVC) had the highest performance for Model 1 (about 87% accuracy). For Model 2 the highest accuracy was considerably slower (77%) and was reached by the Multinomial Naïve Bayes algorithm. Model 3 achieved a similar maximum accuracy (of about 77%) but this time the K-Nearest Neighbors algorithm was the most accurate.

In the end, the aim of our study was to identify potential predictive markers in NET patients. The number of markers in our dataset (also known as features in ML) was much smaller than the number of patients. Therefore, from the ML perspective we did not expect to identify too many of them as redundant. Table 2 summarizes the features that were selected for the three studied models. In the end, the feature that was marked as redundant in all three models was the metastatic site (lung). Two models indicated the exclusion of the following features: metastatic site(bone), grade and type of SSA. This is not to say that these features were irrelevant for the studied prediction but possibly the inclusion of features that were included in the model already made the contribution of the excluded features redundant.

It would be important to further validate this approach by applying it to another similar dataset but at this point and to the best of our knowledge no such dataset is available. To mitigate this, the model was cross-validated with ten randomly chosen seeds during the hyperparameter estimation. The results of these runs can be found in the Appendix A.

## 4. Materials and Methods

### 4.1. Patient Population and Methods

A total of 74 adult patients diagnosed with GEP NET and treated with an SSA (octreotide LAR and lanreotide depot) as a first-line therapy at the Clinical Oncology Unit, AOU Careggi-Firenze and at the European Institute of Oncology, IEO, Milano (Italy) were included in this retrospective analysis. The selection criteria were an histologically confirmed NET diagnosis from a gastrointestinal or pancreatic origin, advanced disease not suitable for radical surgery or residual disease after surgery treated with an SSA (octreotide LAR 30 mg q28 or lanreotide 120 mg q28) as a first-line therapy. The tumors were classified according to the World Health Organization (WHO) classification and the novel TNM classification/G grading system. The Ki67 proliferative index was expressed as a percentage based on the count of Ki67-positive cells in 2000 tumor cells in areas of the highest immunostaining using the MIB1 antibody. All of the patients had computed tomography (CT) scans and somatostatin receptor scintigraphy (SRS) at the time of the initial evaluation and the assessment of the therapeutic outcome was usually repeated every six months unless clinical conditions required shorter intervals. The main endpoint was PFS, defined as the interval between the diagnosis and the time of the first progressive disease (PD) or patient death if it occurred before the documented PD.

### 4.2. Pre-Processing and Oversampling

All data were prospectively collected at the center where the patients had been treated. A unique computerized datasheet was created and all of the data regarding demographic, clinic and pathologic features were retrospectively analyzed.

The continuous variables were transformed into binary variables choosing proper cutoffs and normalization was applied. The dataset imbalance also needed to be analyzed and fixed with appropriate methods. Indeed, when working with an imbalanced dataset, classifiers are biased towards the majority class and tend to highly misclassify the minority class instances. This effect is particularly critical in small datasets [43]. A broadly used rebalancing method is the synthetic minority oversampling technique (SMOTE) introduced in 2002 by Chawla et al. [44]. SMOTE performs oversampling of the minority class by creating synthetic examples based on the nearest neighbors of each example of the minority class. The process for the creation of synthetic examples is as follows:

1. Each example of the minority class is considered and the K-Nearest Neighbors belonging to the same class are identified.
2. A line between the considered example and its K-Nearest Neighbor is drawn;
3. Synthetic examples are randomly generated along those line segments.
4. SMOTE works also for multi-class classification problems [45].

### 4.3. Feature Selection

Feature Selection (FS) is a technique for dimensionality reduction consisting of the selection of a subset of features from the higher dimensional set of initial features. The dimensionality reduction of a dataset can be achieved also through other techniques such as feature extraction and transformation. However, only FS allows for the interpretability of the reduced set of features because it maintains the physical meaning of the initial set of features and this is a particularly crucial point in medical applications [46]. Three categories of FS techniques can be identified: filter, wrapper and embedded methods. Filter methods are based on statistical and mathematical tests and are independent from the classifier (e.g., a chi-squared test, ANOVA). Wrapper methods select the most relevant features by testing different subsets in a classification task and then selecting the subset giving the best performance with the tested classifier (e.g., forward selection, backward selection, recursive feature elimination (RFE)). Finally, embedded methods are algorithms that incorporate the FS phase into their learning process (e.g., Lasso regularization [47]). Wrapper methods have the highest computational cost and filter methods have the lowest one. From the analysis of the most recent literature, a new approach for feature selection has been proposed by Gupta et al. [48].

In this work, six different FS methods were applied and a scoring system was developed to select the most relevant features in each of the three proposed classification models. The F-score, mutual information (MI), RFE with a support vector machine (SVM), RFE with logistic regression (LR), RFE with a random forest (RF) and Lasso regularization were used. After performing FS, a score was assigned to each feature based on the number of times it was selected by the six FS techniques in a way similar to the one used by Amin et al. [49]. The final subset of features was obtained by choosing a threshold value for the score. Only the features with a score higher than the threshold were selected and used for the classification task. FS was performed for each one of the three proposed classification models.

### 4.4. ML Algorithms

Many different algorithms can be used alone or in combination to perform automated data analyses. In this section are briefly described the ones that were tested on our dataset seeking the best performances.

- Logistic Regression (LR): this algorithm falls in the family of statistical models. They are diffusely used in ML to predict the risk of developing a certain disease. Although this method models the probability of an output given an input and therefore should not be properly considered as a classifier, it can still be profitably used as such by setting cutoff thresholds [50].
- Decision Tree (DT): this is a structure similar to a flowchart where each internal node holds a test linked through arches (outcome of tests) to other nodes. The children nodes, or “leaves”, represent decisions or classes. DTs are often used in ensemble methods [51], techniques that combine multiple models or algorithms to achieve better predictive performances. A recent evolution is represented in the C5.0 algorithm, which includes feature selection and reduced pruning errors [52,53].
- Random Forest (RF): introduced by Breiman in 2001 [54], it is an ensemble method widely used also in the field of bioinformatics, metagenomics and genomic data analysis [55]. It is a combination of several algorithms for classification or regression, providing enhanced performances and gaining the predictive power of a single DT [56]. The final prediction is obtained as the average or the majority of the estimations from the single DTs. RF shows sound performances and simplified parameter tuning [57].
- Support Vector Machine (SVM): SVMs are often the chosen algorithm thanks to their excellent performance as supervised binary classifiers. They were first introduced by Boser et al. [58]. The binary classes of training data are represented by two subsets (’regions’) of features. This is done by using a linear hyperplane of equation [59]:
wtx + b = 0.
(1)

The parameters in the above Equation (1) come from a training process aimed at optimizing the geometric margin between classes. A “linear SVM” makes use of an elementary hyperplane.

- Naïve Bayes (NB): grounded on the well-known Bayes’ theorem, these probabilistic classifiers have been used in ML since the very beginning and are still often used in clinical decision support systems for their neatness.
- Multinomial Naïve Bayes (MNB): an NB variation with the features representing the frequencies with which a few events have been generated by a multinomial distribution.
- K-Nearest Neighbors (k-NN): an object is ranked by the majority of its neighbors’ votes. K is a small positive integer. If K = 1 then the object is assigned to its neighbor’s class. Typically, for binary classifications, K is not even to avoid finding situations of equality. This method can also be used for regression techniques by assigning to the object the average of the values of the K closest objects. A drawback is due to the predominance of the classes with more objects. This can be compensated with weighing techniques based on distance.
- Gradient Boosting (GB): this produces a predictive model in the form of a set of weak predictive models, typically DTs. It constructs a model similar to boosting methods and generalizes them allowing the optimization of an arbitrary differentiable loss function. Boosting algorithms are views as iterative descending functional gradient algorithms, optimizing a cost function over a space function pointing to a direction with a negative gradient.
- Extremely Randomized Tree Classifier: based on the idea that randomized DTs show a performance as good as classical ones. In the extreme case, fully randomized trees are built whose structures are independent of the output values of the learning sample [60]. This approach provides good accuracy and computational efficiency.
- Multi-Layer Perceptron (MLP): this is an artificial neural network model, mapping sets of input data into a set of appropriate output data. A direct graph is made up of multiple layers of nodes, each fully connected to the next. The nodes or ’neurons’ are provided with a non-linear activation function. If compared with a traditional standard perceptron, MLPs can distinguish data that are not linearly separable [61].

We decided to run all of the algorithms with their default parameter settings first. For the algorithms that exhibited the highest accuracy in this first phase, the hypertuning of parameters was performed.

### 4.5. ML Performance Measures

The metrics used to assess the performance of the ML algorithms are introduced in the following section. They are accuracy, precision, recall and F1-score [62].

- Accuracy: this is a widely used method for assessing how effective one classifier is in predicting the correct classes. It is defined as the sum of all of the true positives (TPs) and true negatives (TNs) divided by all samples, TP + TN + false positives (FPs) + false negatives (FNs).
  (2) $$Accuracy=\frac{TP+TN}{TP+TN+FP+FN}$$
- Precision: in a classification task, the precision is, for a class, defined as TP divided by the total number of elements labeled as positives (i.e., TP + FP). In a binary classifier, this parameter can be also called sensitivity.
  (3) $$Precision=\frac{TP}{TP+FP}$$
- Recall: this is defined as the number of TPs divided by the total amount of “real” positives that includes the TP and the FN.
  (4) $$Recall=\frac{TP}{TP+FN}$$
- F1-score: this is a score computed as the harmonic mean of precision and recall. Its best value is 1, meaning perfect precision and recall.
  (5) $$F1\text{-}score=\frac{TP}{TP+\frac{1}{2}\left(FP+FN\right)}$$

### 4.6. ML Validation

There are a few major ways that we could validate the ML models. The simplest way was to hold out a portion of the data, develop the ML model on the rest of the data and validate the model with the held out data. This approach can be very dependent on the data selected for the validation and the performance of the model and thus may vary greatly. The literature [63] is unanimously in agreement in preferring improved validation, called cross-validation. During the cross-validation, the model is tested repeatedly to ensure there is no overfitting (a risk that the algorithm learns to classify only that particular dataset with a reduced ability to generalize). In our paper we used a 10-fold cross-validation. This meant that samples were divided into ten subgroups (stratified per class). Ten different models were then developed and in each model one subclass was used for the validation while the remaining samples were the training data. The performance scores reported were the average scores over all of the ten models.

### 4.7. Hyperparameter Tuning

Hyperparameter tuning was performed utilizing the GridSearchCV function from Python’s sklearn package. To ensure cross-validation, a repeated stratified k-fold with 10 folds was used. The whole process was repeated with 10 different random seeds. Table A3 summarizes all of the parameters and their respective values that were used during this process.

### 4.8. Workflow

**Figure 3:**
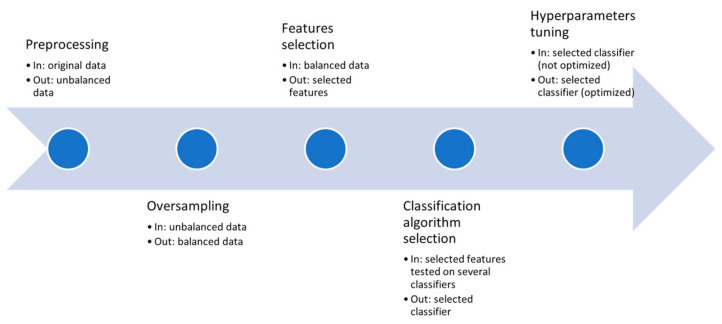
Flowchart of the workflow followed in this study.

Figure 3 is a flowchart that explains the workflow adopted in this study. In the initial study, 74 patients were enrolled and, after data cleaning, 70 were included in the final dataset for classification. Pre-processing was applied consisting of normalization, binarization of Boolean features and an imbalance analysis. Oversampling was then introduced to fix the imbalance and FS was performed. The oversampled data and the selected features were used as input data for the classifiers; their performance was compared and the best performing algorithms were further optimized. The final performance was the highest possible.

The described workflow was used for each of the three models. The third model, having three classes as the possible outcome, was a multi-class problem. In this case, a “one-vs.-all” strategy was adopted both for FS and classification.

## 5. Conclusions

Summarizing, we have documented for the first time that the ML techniques provided a predictive model, which could potentially be used to differentiate prognostic groups in patients with an advanced NET and treated with an SSA. Consistent with previous literature, the predictive factors identified in our study may be useful when stratifying patients with a NET in further studies.

In detail, we studied if we could develop a reliable ML classifier that could predict (based on the value of other markers) if a patient would progress or not within 12 or 18 months. We developed three such models (that achieve accuracy between 77–87%). Furthermore, we identified the set of markers that were redundant in our analysis meaning these markers were not necessary to be considered in order to achieve the maximum classifier accuracy. We declare that the excluded markers were not necessarily unimportant for the prediction of progression but the included markers might have already captured their influence.

The accuracy of the classifier could be further improved by adding information about a greater number of patients to the dataset. Adding new markers or improving the accuracy of measuring the used markers could also bring an additional insight into the two studied groups of patients and raise the accuracy of the classifier. Finally, our study suggested that ML was a promising model to address the value of clinical or biological factors in terms of the prediction of the response/efficacy to antitumor treatments in GEP NETs.

In conclusion, what we discovered in this research confirmed that of previous studies about GEP NETs markers. The amount of work needed to establish those markers is far greater when non-computational methods are used. By using the computational methods, we were able in just one study to evaluate the influence of multiple markers. This approach is very useful not only for the validation of the previous findings but also as a tool that can help prioritize the studies of the individual markers.

Finally, we focused our efforts on the treatment because currently there are no predictive biomarkers of SSA efficacy in patients with NETs. We have documented, for the first time, that the factors that mainly influenced progression-free survival (PFS) included age, the number of metastatic sites and the primary site. Those three features were identified by all three studied models. In addition, two models indicated the following features as important: adverse events G3-G4, sex, Ki67, metastatic site (liver), functioning NET, the primary site and the stage.

These innovative results open a new perspective and confirm that studies focusing on these factors but including more patients and from different countries (international studies) would be important for future studies as NETs rarely result in malignancies.

## Figures and Tables

**Table 1:** Meaning of outcomes in each model.

Model	Outcome 1	Outcome 2	Outcome 3
1	0 (progression within 12 months)	1 (progression free after 12 months)	NA
2	0 (progression within 18 months)	1 (progression free after 18 months)	NA
3	0 (progression within 12 months)	1 (progression between 12 and 18 months)	2 (progression free after 18 months)

**Table 2:** Selected features for each model and their scores.

Model 1			Model 2			Model 3		
Features		Score	Features		Score	Features		Score
1	GENDER	6	1	AGE70	6	1	NET	6
2	PinSITE	6	2	NET	6	2	PinSITE	6
3	NMETA	6	3	PinSITE	6	3	NMETA	6
4	BONEMETA	6	4	Ki67	6	4	SSA	6
5	Ki67	6	5	SSA	6	5	PSITE	5
6	LIVERMETA	4	6	LIVERMETA	4	6	Ki67	5
7	AGE	3	7	AEG3-4	4	7	AGE70	4
8	PSITE	3	8	AGE	3	8	BONEMETA	3
9	STAGE	3	9	NMETA	3			
10	GRADE	3						
11	AEG3-4	3						

**Table 3:** Performance of different ML algorithms. Performance of different ML algorithms. For Model 3 precision, recall and f1-score are calculated using macro.

Algorithm	Accuracy	Precision	Recall	F1-Score
	Average (St.Dev.)	Average (St.Dev.)	Average (St.Dev.)	Average (St.Dev.)
Logistic regression				
Model 1	79.5% (11.8%)	86.5% (18.3%)	74% (16.5%)	77.9% (12.7%)
Model 2	70.4% (20.1%)	76% (23%)	66.5% (25.3%)	67.7% (21.6%)
Model 3	75.4% (22.8%)	78.7% (18.6%)	75.5% (22.8%)	75.1% (22.7%)
Random Forest				
Model 1	84.4% (14%)	88.1% (18.7%)	81.3% (14.8%)	83.7% (14.5%)
Model 2	70.3% (21.1%)	70.3% (22.6%)	66.5% (19.8%)	69.4% (18.3%)
Model 3	73.8% (27.1%)	75% (28%)	75.3% (25%)	75.2% (26.2%)
SVC				
Model 1	87.1% (12.3%)	88.6% (17.5%)	88.7% (9.3%)	87.6% (11.2%)
Model 2	74% (17.6%)	74.5% (21.2%)	74% (20%)	73.5% (18.8%)
Model 3	76.2% (25.2%)	77.5% (24.3%)	76.2% (25.2%)	75.3% (26.1%)
Gaussian Naïve Bayes				
Model 1	51% (4.6%)	25% (40.3%)	5.7% (8.7%)	9% (13.9%)
Model 2	48.9% (4.2%)	4.3% (12.9%)	7.5% (22.5%)	5.5% (16.4%)
Model 3	55.9% (12.7%)	45% (22%)	55.2% (14.4%)	46% (15.4%)
K-Nearest Neighbors (3)				
Model 1	84.3% (14.7%)	87.4% (18.2%)	83.3% (13%)	84.4% (13.5%)
Model 2	71.3% (14.1%)	72.6% (18.6%)	71% (21.1%)	70% (15.7%)
Model 3	65.3% (22.5%)	69.6% (20.1%)	65.5% (22.5%)	64.4% (22%)
Decision Trees				
Model 1	84.5% (16.3%)	89% (18.5%)	79.7% (21.3%)	82% (16.5%)
Model 2	69% (18.5%)	66.8% (30.9%)	58% (30.2%)	67.6% (23.7%)
Model 3	73.8% (21.5%)	77.4% (18.6%)	74% (21.9%)	73.5% (21.6%)
Gradient Boosting				
Model 1	83.5% (15.6%)	85.5% (20%)	83.3% (18.1%)	83.1% (16.6%)
Model 2	59.9% (23.8%)	61% (31%)	60% (32.3%)	57.4% (29.2%)
Model 3	76.1% (22.4%)	76.1% (24.1%)	76% (22.8%)	75.2% (23.7%)
Extra Trees				
Model 1	85.2% (10%)	89.5% (16.3%)	85.3% (10.6%)	85.1% (9.2%)
Model 2	65.6% (18.2%)	66.3% (21.3%)	69% (19.1%)	63% (19.8%)
Model 3	72.2% (24.3%)	73.7% (25.8%)	73% (25.2%)	70.5% (26.4%)
MultinomialNB				
Model 1	82.2% (12.7%)	86.8% (15.9%)	81.7% (20.2%)	81.2% (13.8%)
Model 2	76.1% (13.1%)	73.6% (14.4%)	78.5% (22.6%)	75% (16.3%)
Model 3	68.3% (14.7%)	71.5% (14.8%)	67.8% (14.5%)	65.9% (14.7%)
MLP				
Model 1	86.9% (9.3%)	86.9% (17.1%)	86.7% (8.8%)	85% (12.4%)
Model 2	67.9% (20.4%)	74.5% (21.7%)	66.5% (25.3%)	64.6% (21.7%)
Model 3	76.9% (22.3%)	78% (23.7%)	78.5% (22.6%)	78.2% (22.4%)

**Table 4:** The average accuracies of algorithms before and after the hyperparameter tuning.

Algorithm	Initial Accuracy (Default Parameter Setting)	Accuracy after Hyperparameters Tuning (Average and Std)
Logistic regression		
Model 1	79.5%	83.6% (1.08%)
Model 2	70.4%	72% (0.9%)
Model 3	75.4%	76.3% (0.3%)
MultinomialNB		
Model 1	82.2%	82.5% (0.8%)
Model 2	76.1%	77.3% (0.7%)
Model 3	68.3%	70% (0.5%)
MLP		
Model 1	86.9%	86.7% (0.7%)
Model 2	67.9%	71.7% (1.03%)
Model 3	76.9%	77.7% (0.6%)
SVC		
Model 1	87.1%	86.2% (0.5%)
Model 2	74%	73.6% (1.33%)
Model 3	76.2%	77.4% (0.8%)
KNNeighbors		
Model 1	84.3%	85.2% (0.7%)
Model 2	71.3%	72.9% (1.15%)
Model 3	65.3%	77.3% (0.96%)

## Data Availability

Data available on request to the corresponding author.

## Appendix A. Tables

**Table A1:** Dataset.

Patients Number	74	100%
Age (years)		
Median	68	
Range	24–90	
>70	57	77%
≥70	17	23%
Sex		
M	45	61%
F	29	39%
Performance status (ECOG)		
0–1	73	99%
2	1	1%
Primary site		
Pancreas	30	41%
Gastrointestinal	44	59%
Functioning NET		
Yes	22	30%
No	52	70%
Grade		
G1	26	35%
G2	46	62%
NA	2	3%
Ki67		
<2%	19	26%
2–20%	51	69%
≥20%	2	2.5%
NA	2	2.5%
Stage		
Locally advanced	2	3%
Metastatic	72	97%
Primary on site		
Yes	31	42%
No	43	58%
Number of metastatic sites		
0	2	3%
1	46	62%
>1	26	35%
Metastatic sites		
Liver	63	85%
Lung	5	7%
Bone	3	4%
Type of SSA		
Lanreotide	34	46%
Octreotide LAR	40	54%
Adverse events G3-G4		
Yes	1	1%
No	73	99%
PFS > 12 months		
Yes	53	72%
No	17	23%
NA	4	5%
PFS > 18 months		
Yes	43	58%
No	27	37%
NA	4	5%

**Table A2:** The original count of samples for each studied model.

Model	12 Month Progression	18 Month Progression	No Progression
1	17	NA	53
2	NA	27	43
3	17	10	43

**Table A3:** The parameters and their values used in the hyperparameter tuning process.

Algorithm	Parameter	Parameter Values
Logistic regression	solvers	‘newton-cg’, ‘lbfgs’, ‘liblinear’
	penalty	I2
	c_values	100, 10, 1.0, 0.1, 0.01
Multinomial NB	alpha	1, 0.9, 0.8, 0.7, 0.6, 0.5, 0.1, 0.01, 0.001, 0.0001, 0.00001
	fit_prior	True, False
	class_prior	None, (0.5, 0.5), (0.4, 0.6), (0.45, 0.55), (0.6, 0.4), (0.1, 0.9), (0.2, 0.8)
MLP	hidden_layer_sizes	(50, 50, 50), (50, 100, 50), (100)
	activation	‘tanh’, ‘relu’
	solver	‘sgd’, ‘adam’
	alpha	0.0001, 0.05
	learning_date	‘constant’, ‘adaptive’
SVC	C	0.1, 1, 10, 100, 1000
	gamma	1, 0.1, 0.01, 0.001, 0.0001
	kernel	rbf
K-Nearest Neighbors	n_neighbors	3, 5, 11, 19
	weights	uniform, distance
	metric	‘euclidean’, ‘manhattan’
